# Targeting circular RNA-ZRANB1 for therapeutic intervention in retinal neurodegeneration

**DOI:** 10.1038/s41419-018-0597-7

**Published:** 2018-05-10

**Authors:** Jia-Jian Wang, Kun Shan, Bai-Hui Liu, Chang Liu, Rong-Mei Zhou, Xiu-Miao Li, Rui Dong, Shu-Jie Zhang, Sheng-Hai Zhang, Ji-Hong Wu, Biao Yan

**Affiliations:** 1 Eye Institute, Eye & ENT Hospital, Shanghai Medical College, Fudan University, Shanghai, China; 2 Shanghai Key Laboratory of Visual Impairment and Restoration, Shanghai, China; 3 Department of Pediatric Surgery, Children’s Hospital of Fudan University, Shanghai, China; 4 The Fourth School of Clinical Medicine, Nanjing Medical University, Nanjing, China

## Abstract

Glaucoma is a neurodegenerative disease characterized by retinal ganglion cell (RGC) loss, optic disc excavation, and progressive visual field loss. Direct or indirect ameliorating retinal neurodegeneration is a promising therapeutic therapy for glaucoma. Circular RNAs (circRNAs) are a class of covalently closed circular RNA transcripts and have emerged as potential regulators in several neurodegenerative diseases. In this study, we show that cZRANB1 expression is significantly upregulated in retinal neurodegeneration induced by glaucoma. cZRANB1 knockdown decreases retinal reactive gliosis, glial cell activation, and facilitates RGC survival in vivo. cZRANB1 knockdown directly regulates Müller cell function and indirectly regulates RGC function in vitro. cZRANB1 acts as miRNA sponge to regulate Müller cell function through cZRANB1/miR-217/RUNX2 network. Intervention of cZRANB1 expression would become an effective strategy for treating retinal neurodegeneration.

Glaucoma is recognized as a retinal neurodegenerative disease characterized by retinal ganglion cell (RGC) loss, optic disc excavation, and progressive visual field loss^1^. It is a major cause of vision impairment and irreversible blindness in the world^2^. Previous studies have shown that the occurrence and development of glaucoma is associated with elevated intraocular pressure (IOP), increased oxidative stress, aging, glutamate neurotoxicity, endoplasmic reticulum stress, and mutations in susceptibility genes^3, 4^, suggesting that the pathogenesis of glaucoma is multifactorial and highly complex.

Currently, the therapeutic methods for glaucoma mainly include surgery and pharmaceuticals. They partially delay or halt the progression of glaucoma and rescue the visual field, as well as RGC survival^5^. However, despite effective medical and surgical therapies to reduce IOP, progressive vision loss among glaucoma patients is common^6^. Thus, it is still required to further elucidate the pathogenesis of glaucoma.

Circular RNAs (circRNAs) are a class of covalently closed circular transcripts generated by the back-splicing of pre-mRNAs. They play important roles in several biological progresses, including cell proliferation, migration, apoptosis, and differentiation^7–9^. Increasing studies have shown that circRNA dysregulation has been implicated in several neurodegenerative diseases, such as Alzheimer’s disease (AD), Parkinson’s disease (PD), and amyotrophic lateral sclerosis (ALS)^10^. The retina is the extension of the brain and a part of central nervous system (CNS). Glaucoma is usually recognized as a retinal neurodegenerative disease^11^. We speculate that circRNAs may play an important role in the pathogenesis of glaucoma.

In this study, we investigated the expression pattern and the role of cZRANB1 in glaucoma-induced retinal neurodegeneration. cZRANB1 (hsa_circ_0000268) is located at chr10:126631025–126631876. Its expression is highly conserved across human, mouse, and rat. We reveal that cZRANB1 expression is significantly upregulated in the glaucomatous retinas. cZRANB1 knockdown significantly decreases retinal reactive gliosis, inhibits Müller cell activation, and facilitates RGC survival. cZRANB1 knockdown directly regulates Müller cell function but indirectly regulates RGC function in vitro. Intervention of cZRANB1 expression would become a promising therapeutic strategy for retinal neurodegeneration.

## Results

### cZRANB1 expression is significantly upregulated during glaucoma-induced retinal neurodegeneration

**Fig. 1:**
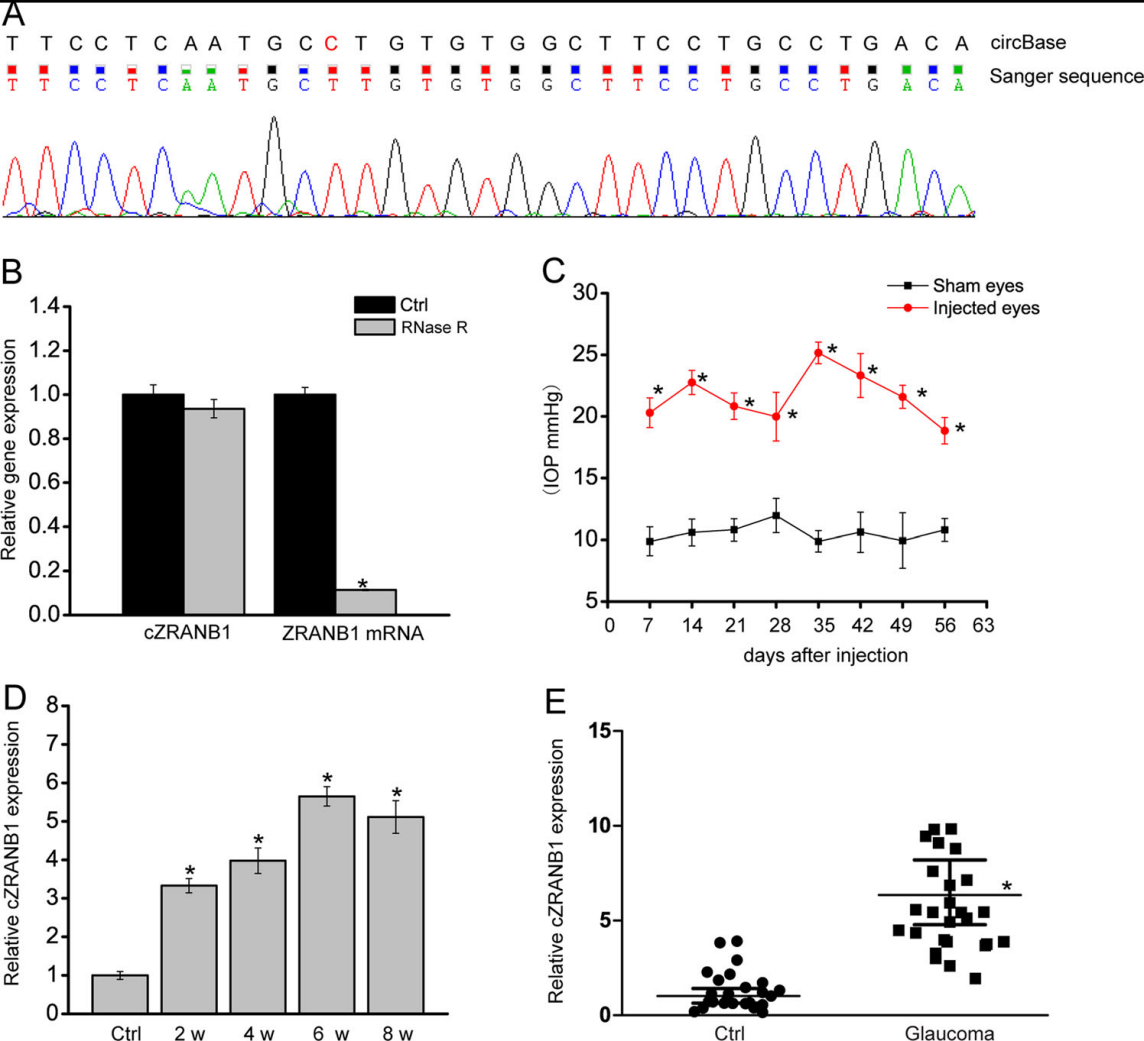
cZRANB1 expression is significantly upregulated during glaucoma-induced retinal neurodegeneration. **a** The amplified product of cZRANB1 using the total RNAs of rat retinal lysates was sent for Sanger sequencing, and then compared with mouse cZRANB1 sequence as  recorded in circBase. **b** Total RNAs from rat retinas were digested with RNase R followed by qRT-PCRs detection of cZRANB1 expression. ZRANB1 mRNA was detected as the RNase R-sensitive control (*n* = 4). **c**, **d** Glaucoma was induced by anterior chamber injection of microbeads. IOP levels in the eyes injected with PBS buffer (sham eyes) and microbeads (injected eyes) were shown (**c**, *n* = 5). qRT-PCRs were conducted to detect cZRANB1 expression in the retinas of rats after microbead injection at the indicated time points (**d**, *n* = 5). **e** The aqueous humor was obtained fromglaucoma patients (*n* = 25) and cataract patients (*n* = 25) without ocular neurodegenerative diseases. qRT-PCRs were conducted to detect cZRANB1 expression. All data were from at least three independent experiments

We first determined whether cZRANB1 was expressed in rat retinas by Sanger sequencing. The result showed that the sequence of amplified product of cZRANB1 was nearly  in accordance with mouse cZRANB1 sequence (mmu_circ_0013957) as previously reported in circBase (Fig. 1a). cZRANB1 was resistant to RNase R digestion, whereas linear ZRANB1 mRNA was easily degraded (Fig. 1b).

We built the rat model of glaucoma bychamber injection of microbeads. The injection of microbeads led to a marked elevation in IOP level (20–25 mmHg), about two times higher than normal IOP level (Fig. 1c). IOP level was maintained for another 4 weeks with a second microbead injection. Meanwhile, we detected retinal cZRANB1 expression pattern in this model. Elevated IOP led to a marked increase in cZRANB1 expression (Fig. 1d). Moreover, cZRANB1 expression was significantly upregulated in the aqueous humor from the patients with primary open-angle glaucoma (Fig. 1e).

### cZRANB1 knockdown inhibits retinal reactive gliosis and facilitates RGC survival in vivo

**Fig. 2:**
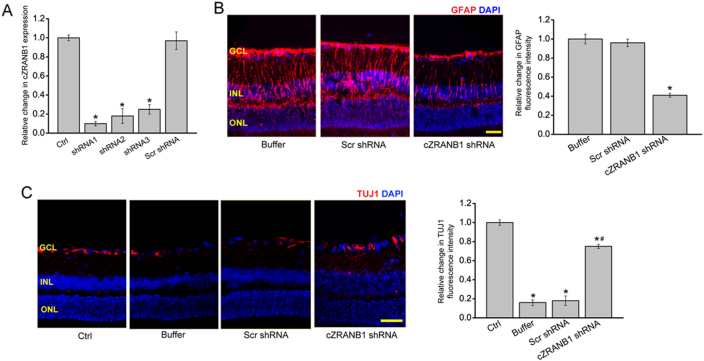
cZRANB1 knockdown reduces retinal reactive gliosis and facilitates RGC survival in vivo. **a** Eight weeks after microbead injection, the rat retinas were injected with 5 μl scramble (Scr) shRNA, cZRANB1 shRNA-1, cZRANB1 shRNA-2, cZRANB1 shRNA-3, or left untreated (Ctrl) for 7 days. qRT-PCRs were conducted to detect cZRANB1 expression (*n* = 5, *P < 0.05 vs. Ctrl group). **b** Eight weeks after microbead injection, retinal slices were stained with GFAP to detect retinal reactive gliosis in PBS buffer-, Scr shRNA-, and cZRANB1 shRNA1-injected glaucomatous retinas (*n* = 5, *P < 0.05 vs. buffer group). **c** Eight weeks after microbead injection, retinal slices were stained with TUJ1 to detect RGC survival in healthy retina (Ctrl) and PBS buffer-, Scr shRNA-, and cZRANB1 shRNA1-injected glaucomatous retinas (*n* = 5, *P < 0.05 vs. Ctrl group). Scale bar, 100 µm. GCL ganglion cell layer, INL inner nuclear layer, ONL outer nuclear layer. ** indicates significant difference compared with the corresponding control group

We designed three different short hairpin RNA (shRNAs) for cZRANB1 knockdown. All of them could significantly downregulate cZRANB1 expression (Fig. 2a). We performed protein immunofluorescence to determine the role of cZRANB1 in glaucoma-induced retinal neurodegeneration in vivo. Compared with microbead injection-induced retinal neurodegenerative retina, cZRANB1 knockdown significantly inhibited reactive gliosis as shown by decreased Glial fibrillary acidic protein (GFAP) staining and facilitated RGC survival as shown by increased TUJ1 staining (Figs. 2b, c). Retinal slices were also immunolabeled with the marker proteins, including Calretinin (ganglion cells and amacrine cells), Rhodopsin (Rod and cone photoreceptor), and protein kinase Cα (PKCα; bipolar cells). Compared with microbead injection-induced glaucomatous retina, cZRANB1 knockdown increased the staining signaling of calretinin-labeled cells in the ganglion cell layer (GCL), but did not further affect the staining signaling of calretinin-labeled cells in the inner nuclear layer (INL) (Fig. S1A). We also showed that cZRANB1 knockdown did not affect the staining signaling of amacrine cell, rod and cone photoreceptor, and bipolar cell (Fig. S1A-C).

### cZRANB1 directly regulates Müller cell but indirectly regulates RGC function in vitro

**Fig. 3:**
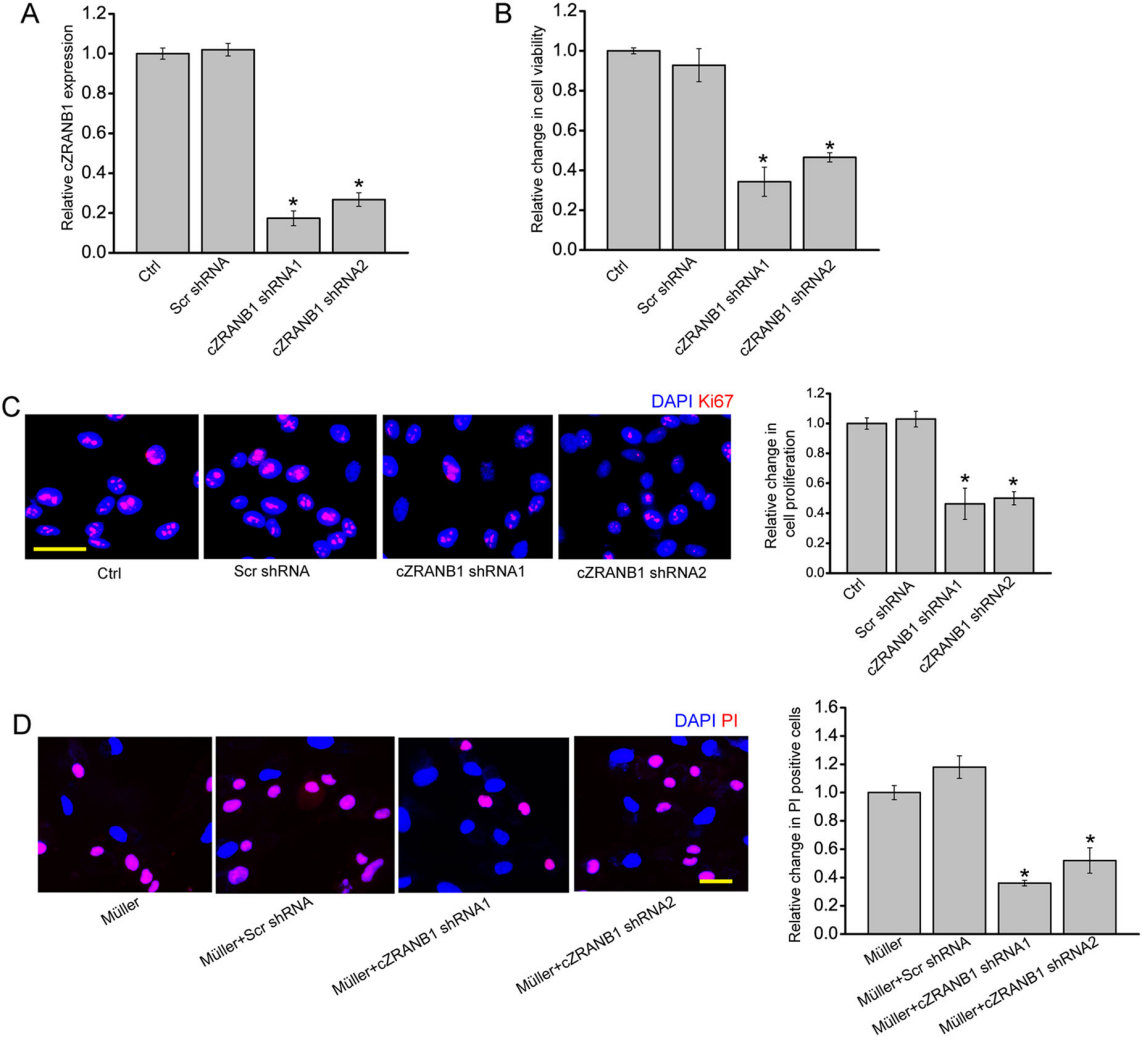
cZRANB1 knockdown directly regulates Müller cell but indirectly regulates RGC function in vitro. **a** Müller cells were transfected with scramble (Scr) shRNA, cZRANB1 shRNA1, cZRANB1 shRNA2, or left untreated (Ctrl) for 24 h. qRT-PCR assays were conducted to detect cZRANB1 expression (*n* = 4, *P < 0.05 vs. Ctrl). **b** The viability of Müller cells was detected by MTT method. The data were expressed as the relative change compared with Ctrl group (*n* = 4, *P < 0.05 vs. Ctrl). **c** Ki67 staining was used to detect Müller cell proliferation (*n* = 4, *P < 0.05 vs. Ctrl group). Scale bar: 20 μm. **d** RGCs were co-cultured with wild-type Müller cells (Ctrl), Scr shRNA-transfected Müller cells, cZRANB1 shRNA1-transfected Müller cells, or cZRANB1 shRNA2-transfected Müller cells, and then treated with H_2_O_2_ (100 μm) for 24 h. PI staining and quantitative analysis was performed to detect apoptotic RGCs (*n* = 4, *P < 0.05 vs. Ctrl)

Based on the above-mentioned results, we speculated that cZRANB1 played its role in glaucoma-induced retinal neurodegeneration by targeting Müller cells and RGCs. We then investigated the role of cZRANB1 in vitro. cZRANB1 shRNA transfection obviously decreased cZRANB1 expression in Müller cells (Fig. 3a). MTT assay and Ki67 staining revealed that cZRANB1 knockdown significantly decreased Müller cell viability and proliferation in vitro (Figs. 3b, c). cZRANB1 shRNA transfection significantly decreased cZRANB1 expression in the primary RGCs (Fig. S2A). However, cZRANB1 knockdown did not affect RGC viability and proliferation in vitro (Fig. S2B and S2C). We then employed RGC-Müller co-culture system to investigate whether cZRANB1 knockdown in Müller cells had an indirect effect on RGC function. Mechanisms involved in RGC injury in glaucoma mainly include oxidative injury and glutamate toxicity^4, 6^. RGCs were exposed to H_2_O_2_ or glutamate to mimic oxidative stress and glutamate toxicity stress. propidium iodide (PI) staining revealed that cZRANB1 knockdown in Müller cells significantly reduced the pro-apoptotic effects of Müller cells on RGCs under oxidative stress and glutamate toxicity stress (Fig. 3d and Fig. S2D). Collectively, these results suggest that cZRANB1 directly regulates Müller cell but indirectly regulates RGC function.

### cZRANB1 knockdown affects glial cell activation in vivo

**Fig. 4:**
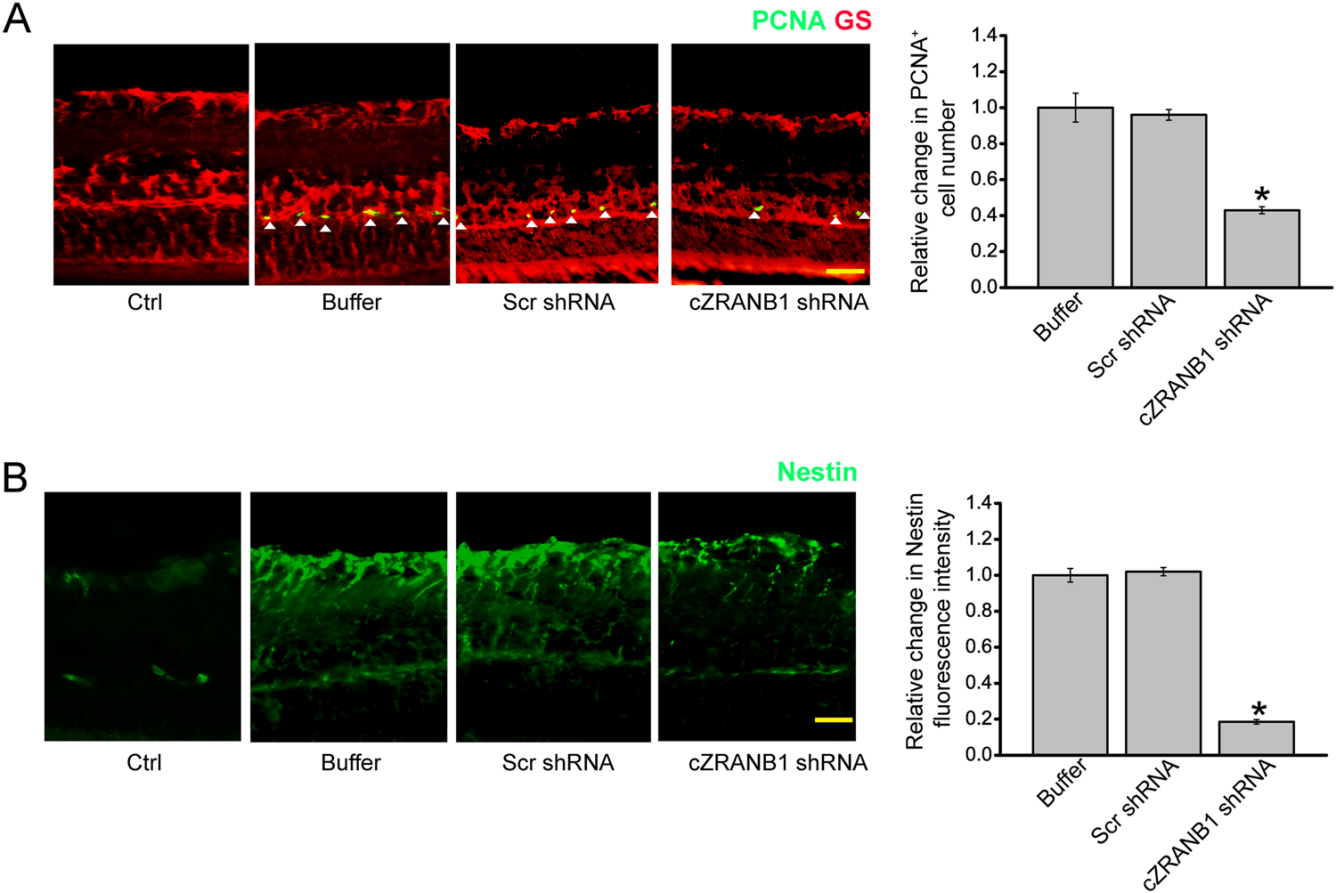
cZRANB1 knockdown affects glial cell activation in vivo. **a**, **b** The rat retinas were injected with 5 μl scramble (Scr) shRNA, cZRANB1 shRNA-1, or PBS buffer for 7 days, and then injected with the microbead for 8 weeks. The retinas only injected with PBS buffer were taken as the control group. Retinal slices were stained with PCNA and GS antibody to detect glial cell proliferation (**a**; *P < 0.05 vs. buffer group; *n* = 5). Retinal slices were stained with Nestin (**b**; *P < 0.05 vs. buffer group; *n* = 5) to investigate the effect of cZRANB1 knockdown on the reactivation of stem and progenitor properties of glial cells. Scale bar, 100 µm. GCL ganglion cell layer, INL inner nuclear layer, ONL outer nuclear layer

Retinal glia usually dedifferentiates and reenters the proliferation cycle in response to stress. We thus investigated whether cZRANB1 knockdown affected the regenerative ability of Müller cells in vivo. We employed Proliferating cell nuclear antigen (PCNA) immunofluorescence staining to detect the proliferation of retinal cells. cZRANB1 knockdown significantly decreased the number of PCNA-positive cells in glaucomatous retinas. Moreover, PCNA staining cells were overlapped with glutamine synthetase (GS) staining (Fig. 4a), suggesting that cZRANB1 knockdown affects Müller glia proliferation in vivo.

The reactivation of stem and progenitor properties of glial cells also promoted us to investigate the effect of cZRANB1 knockdown on the expression of progenitor marker, nestin. Nestin protein localizes mainly in neural progenitor cells or undifferentiated astrocytes^12^. We showed that cZRANB1 knockdown significantly reduced the expression of nestin in glaucomatous retinas (Fig. 4b), suggesting that cZRANB1 knockdown affects the reactivation of stem and progenitor properties of glial cells.

### cZRANB1 acts as a miRNA sponge in Müller cells

**Fig. 5:**
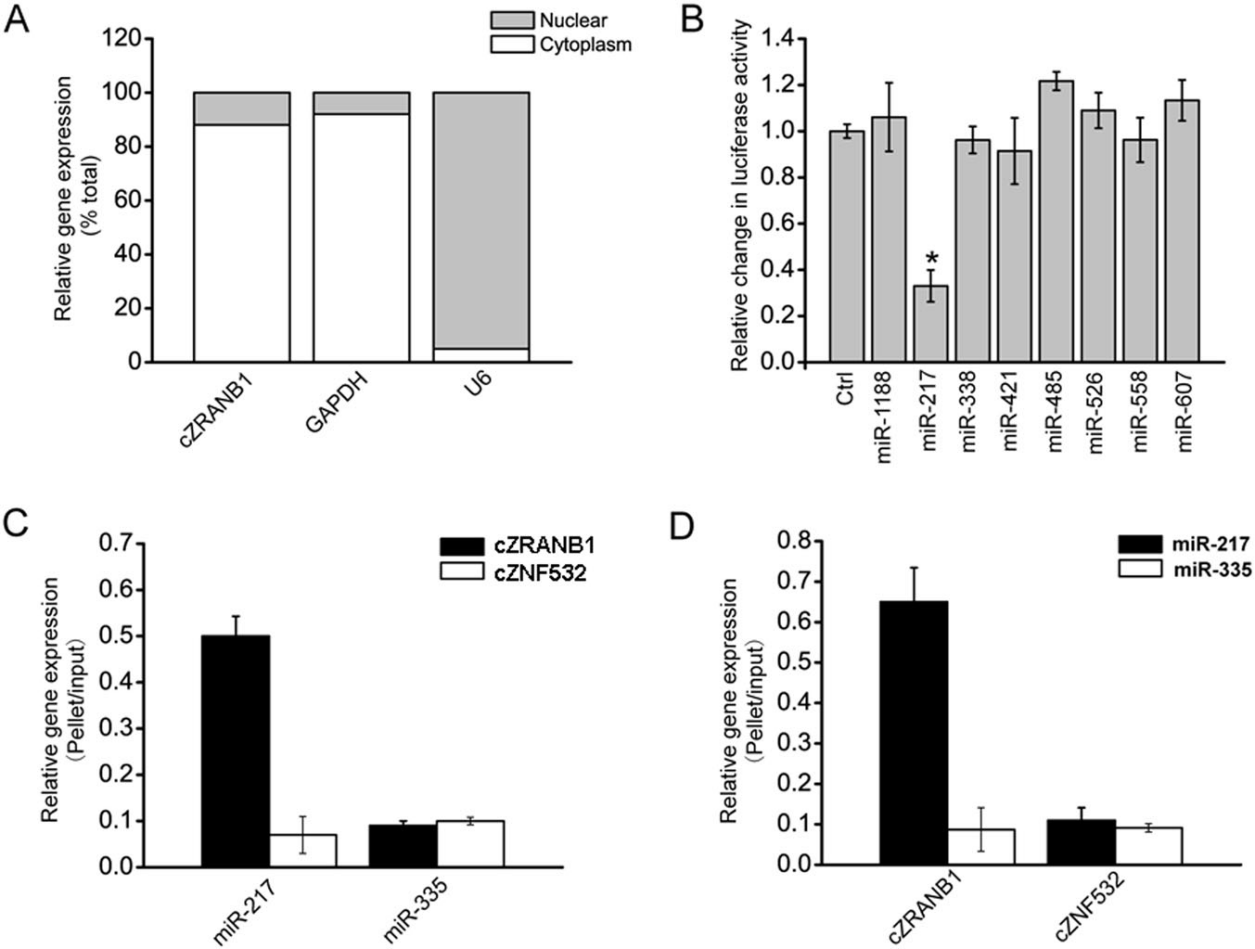
cZRANB1 acts as a miRNA sponge in Müller cells. **a** The expression of nuclear control transcript (U6), cytoplasmic control transcript (GAPDH), and cZRANB1 was detected by qRT-PCRs in the nuclear and cytoplasmic fraction of Müller cell (*n* = 4). **b** The full sequence of cZRANB1 was cloned into the pGL3 Luciferase Reporter to construct LUC-cZRANB1 vector. Müller cells were co-transfected LUC-cZRANB1 with different miRNA mimics. Luciferase activity was detected by the dual luciferase assay at 24 h after transfection (*n* = 4, *P < 0.05 vs. Ctrl group). **c** The 3′-end biotinylated miRNA duplexes were transfected into Müller cells. After the streptavidin capture, the amount of cZRANB1 and cZNF532 (negative control) in the input and bound fractions were detected by qRT-PCRs (*n* = 4). **d** The 3′-end biotinylated cZRANB1 or cZNF532 (negative control) was transfected into Müller cells. After the streptavidin capture, the amount of miR-217 and miR-335 (negative control) in the input and bound fractions were detected by qRT-PCRs (*n* = 4)

Quantitative reverse transcriptase-PCR (qRT-PCR) assays revealed that cZRANB1 was mainly expressed in the cytoplasm of Müller cells (Fig. 5a). Previous study has revealed that cytoplasm-expressed circRNA could regulate cell function by acting as a miRNA sponge^9^. Based on TargetScan and miRana program, we predicted the miRNAs, which could potentially bind to cZRANB1. Luciferase activity assays showed that miR-217 mimic transfection significantly decreased luciferase activity of LUC-cZRANB1 (Fig. 5b). RNA pull-down assays showed that cZRANB1 was greatly enriched in miR-217-captured fraction compared with the negative control, biotinylated miR-335 (Fig. 5c). We also found that miR-217 was greatly enriched in cZRANB1-captured fraction compared with the negative control, biotinylated cZNF532 (Fig. 5d). These results suggest that cZRANB1 acts as a miRNA sponge in Müller cells.

### cZRANB1/miR-217/RUNX2 network is involved in regulating Müller cell function

**Fig. 6:**
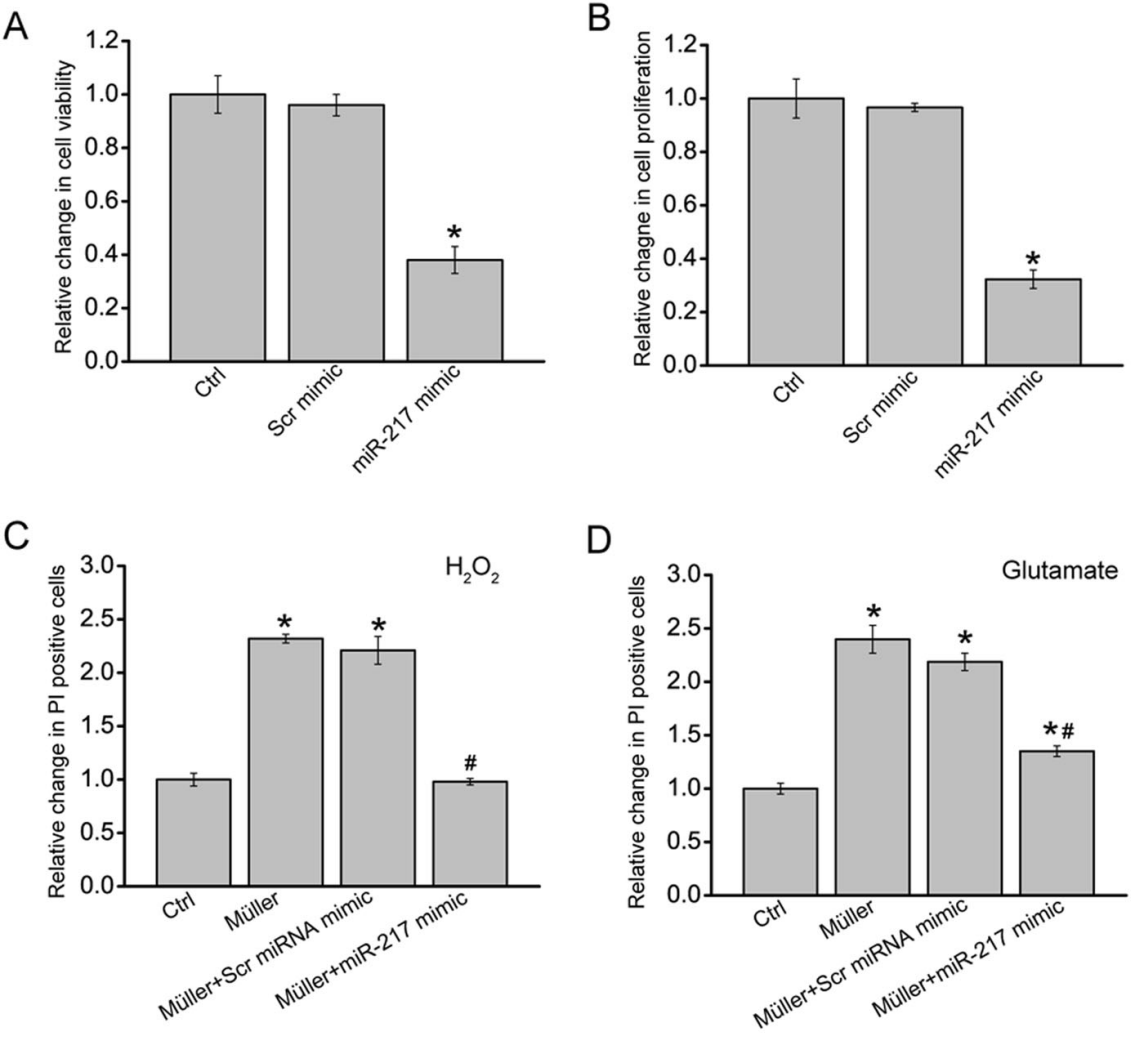
cZRANB1-miR-217-RUNX2 is involved in regulating Müller cell function. **a**, **b** Müller cells were transfected with scramble (Scr) miRNA mimic, miR-217 mimic, or left untreated for 24 h. MTT assays **a** and Ki67 staining **b** was conducted to detect cell viability and proliferation (*n* = 4, *P < 0.05 vs. Ctrl group). Scale bar: 20 μm. **c,** **d** Two days prior to the co-culture, Müller cells were transfected with miR-217 mimic, Scr mimic, or left untreated. At the day of co-culture, Müller cells were cultured on the upper chamber of Transwell. RGCs were cultured on the below chamber. RGCs was cultured alone as the control group (Ctrl). They were then incubated with H_2_O_2_ (**c**, 100 μm) or glutamate (**d**, 3 mM) for 24 h. After the above-mentioned treatments, the upper chamber was removed. PI staining and quantitative analysis was used to detect apoptotic RGCs (*n* = 4, *P < 0.05 vs. Ctrl group; ^#^*P* < 0.05 Müller cell + Scr miRNA mimic vs. Müller cell + miR-217 mimic group). All data were from at least three independent experiments

As miR-217 was sponged by cZRANB1, we then investigated the role of miR-217 in Müller cells. miR-217 mimic transfection significantly decreased Müller cell viability and proliferation (Figs. 6a, b). Müller cell and RGC co-culture and PI staining assays showed that miR-217 mimic transfection in Müller cells obviously attenuated the harmful effects of Müller cells on RGCs in response to H_2_O_2_ or glutamate treatment, which could mimic cZRANB1 knockdown (Figs. 6c, d).

We employed the Targetscan database to predict the target gene of miR-217. One of candidate genes, RUNX2, aroused our interest due to its role in cell differentiation and proliferation^13, 14^. miR-217 mimic transfection significantly reduced RUNX2 expression (Fig. S3A). Luciferase reporter assay further verified the direct regulation of miR-217 on RUNX2 expression (Fig. S3B). We also revealed that cZRANB1 knockdown significantly reduced RUNX2 expression (Fig. S3C). Moreover, cZRANB1 knockdown-mediated inhibition of Müller cell proliferation could be reversed by RUNX2 overexpression (Fig. S3D).

## Discussion

Glaucoma is a leading cause of irreversible vision loss characterized by retinal neurodegeneration, including progressive RGC loss and axon degeneration. Directly or indirectly facilitating RGC survival could delay or halt the progression of glaucoma. Here, we show that cZRANB1 expression is significantly upregulated in glaucoma-induced retinal neurodegeneration. cZRANB1 knockdown significantly decreases retinal reactive gliosis, glial cell activation, and facilitates RGC survival in vivo. cZRANB1 knockdown directly regulates Müller cell function but indirectly regulates RGC function in vitro. This study provides a novel insight into the pathogenesis of glaucoma.

Under the normal condition, glial cells maintain neuronal function by removing extracellular glutamate or producing the growth factors and metabolites. Previous study has shown that glial cell activation occurs in the glaucomatous retinas^15^. The function of glial cell activation is not fully understood. On one side, glial cells detect the elevation of IOP through their activation, and initiates gene regulatory signaling to assist RGCs to resist the insult^16^. cZRANB1 is significantly upregulated during retinal neurodegeneration induced by glaucoma. Increased cZRANB1 expression would cause retinal reactive gliosis and glial cell activation. One the other side, reactive gliosis may potentially exacerbate the glaucomatous process by overexpressing toxins such as reactive oxygen intermediates, proteases, and excitatory amino acids, contributing to RGC degeneration. Typical features of retinal gliosis involve cellular hypertrophy, upregulation of intermediate filament protein expression (e.g., GFAP and Nestin), increased proliferation, loss-of-function, and inflammation^17, 18^. cZRANB1 knockdown significantly decreases retinal reactive gliosis as shown by decreased GFAP and Nestin expression. Moreover, ZRANB1 knockdown reduced glaucoma-induced RGC apoptosis. Thus, ZRANB1 knockdown may decrease the detrimental side of retinal reactive gliosis and exert a beneficial effect on RGC function.

circRNAs play their roles depending on the cell context, such as circRNA abundance, subcellular localization, interacting partners (RNA, DNA, and proteins), dynamic changes in interactions following stimulation, and potential circRNA translation^19, 20^. cZRANB1 is constitutively expressed in the retina. It is mainly expressed in the cytoplasm of glial cells, suggesting a regulatory role at the post-transcriptional level. A majority of studies have shown that circRNAs suppress the expression of miRNAs, thereby increasing the translation and stability of the targets of such miRNAs at the post-transcriptional level^9, 21, 22^. In Müller cells, we show that increased cZRANB1 may sponge and sequester miR-217 upon oxidative stress or glutamate excitotoxicity, releasing miR-217-mediated repressive effects on Müller cell function. The expression of cZRANB1 is significantly upregulated upon stresses. A slight change in gene expression pattern elicited by cZRANB1 upregulation could drive key cellular processes, such as cell proliferation, differentiation, and survival, which govern retinal reactive gliosis.

RUNX2 is a member of the Runx family of transcription factors that plays essential roles during development and adult tissue homeostasis. Alterations of RUNX2 function is associated with several cancers and other human pathologies^14, 23, 24^. It is involved in regulating cell proliferation and differentiation in a variety of cell lineages, such as preosteoblast, endothelial cell, osteosarcoma cell, several tumor cells^25–27^. We show that RUNX2 overexpression could reverse cZRANB1 knockdown-induced inhibitory effects on Müller cell proliferation. Retinal gliosis is usually characterized by increased Müller cell proliferation. During glaucomatous neuropathy, cZRANB1 overexpression becomes a sink for miR-217, and releases the repressive effect of miR-217 on RUNX2 expression. RUNX2 upregulates could contribute to Müller cell proliferation.

Collectively, this study shows that cZRANB1 is potentially involved in retinal neurodegeneration induced by glaucoma. cZRANB1 knockdown reduces retinal reactive gliosis and contributes to RGC survival. cZRANB1 regulates glaucomatous neuropathy through cZRANB1/miR-217/RUNX2 signaling network. This study provides a promising target for treating retinal neurodegeneration.

## Materials and methods

### Ethics statement

All animal studies were performed in accordance with the guidelines of the Care and Use of Laboratory Animals (published by National Institutes of Health) and the ARVO Statement for the Use of Animals in Ophthalmic and Vision Research. All animal experiment was approved by the Animal Care and Use Committee of Eye & ENT Hospital. Clinical samples were used according to the Declaration of Helsinki.

### Cell culture

Retinal glial cells line (rMC-1) and primary RGCs were cultured in Eagle’s medium (Gibco-BRL, Grand Island, NY, USA) in Dulbecco’s modified Eagle’s medium containing 10% fetal bovine serum, 100 U/mL penicillin, and 100 μg/mL streptomycin (Sigma-Aldrich, St. Louis, MO) at 37 °C and 5% CO_2_ in a humidified incubator.

### Induction of glaucomatous rats

Sprague-Dawley (SD, male, weigh 200–250 g) rats were used for the induction of glaucoma through the chamber injection of microbeads. In brief, the animals were anesthetized by intraperitoneal injection of a mixture of xylazine (10 mg/kg) and ketamine (75 mg/kg). After the anesthesia, the cornea was punctured using a 30-gauge needle. One eye was injected with 15-μm polystyrene microbeads, and the other eye was injected with phosphate-buffered saline (PBS) as the control group. The animal was allowed for recovering 24 h before IOP measurement. A second microbead injection was performed 4-week after the injection. IOP measurement was conducted using the digital tonometer between 10 a.m. and 2 p.m. to avoid the effect of circadian rhythm.

### Immunohistochemistry

The eyes of SD rats were isolated and the cornea was cut off along the limbus. The cup-shaped retinas were fixed in 4% paraformaldehyde for 12 h, cryoprotected in 30% sucrose for 12 h, and then embedded in optimum cutting temperature (OCT) medium. For immunohistochemistry, retinal slices were incubated in the blocking solution (5% bovine serum albumin, 0.3% Triton X-100 in PBS) for 30 min at 37 °C. Retinal slices were then incubated with the primary antibodies, including TUJ1 (1:300, Abcam), GFAP (1:200, Abcam), GS (1:200, Abcam), calretinin (1:500, Chemicon), rhodopsin (1:1000, Sigma), PKCα (1:200, Abcam), and nestin (1:100, Santa Cruz) at 4 °C for 24 h. The slices were washed with phosphate buffer saline containing 0.1% Tween 20 (PBST) buffer, and then incubated with the fluorescein isothiocyanate (FITC)- or Cy3-conjugated secondary antibody (Invitrogen) overnight at 4 °C. Finally, the slides were observed and imaged.

### Co-culture assay

Two days prior to the co-culture, Müller cells were transfected with miR-217 mimic, scramble mimic, or left untreated. In order to investigate the crosstalk between Müller cells on RGCs, Müller cells were cultured in a cell co-culturing model, in which Müller cells were cultured on the upper chamber of the Transwell with 0.4 μm pore size. RGCs were cultured on the below chamber of the culturing well. They were then incubated with or without H_2_O_2_ (100 μm) or glutamate (3 mM) for 24 h. After the above-mentioned treatments, the upper chamber was removed. PI staining was conducted to detect the apoptotic RGCs.

### Biotin-coupled miRNA capture

The 3′ end biotinylated miR-217 or control mimic RNA were transfected into Müller cells at the concentration of 20 nM for 24 h. The biotin-coupled RNA complex was pull-downed by incubating the cell lysates with streptavidin-coated magnetic beads (Life Technologies). cZRANB1 amount in the bound fraction was determined by qRT-PCR assays.

### Statistical analysis

Statistical analyses were performed using SPSS software. Results were presented as mean ± SEM. Differences between groups were analyzed using Student’s *t*-test, and comparisons among groups were analyzed using one-way analysis of variance (ANOVA) and Bonferroni’s multiple comparison test. *P* < 0.05 was considered significantly different.

## Electronic supplementary material

Supplementary material
