# Supplementary material for: Targeting circular RNA-ZRANB1 for therapeutic intervention in retinal neurodegeneration
Source: Cell Death Dis. 2018 May 10;9(5):540. doi: 10.1038/s41419-018-0597-7 (PMC5945597; doi:10.1038/s41419-018-0597-7)
Supplement: Supplementary file 1 — Supplementary material [file 41419_2018_597_MOESM1_ESM.pdf]

Supplementary figure legend

Figure S1: cZRANB1 knockdown does not affect the signaling of retinal amacrine cell, photoreceptor, and bipolar cell in vivo

Eight weeks after microbead injection, retinal slices were stained with the marker proteins, including Calretinin, Rhodopsin, and PKCα. Quantitative analysis showed that cZRANB1 knockdown did not affect the signaling of retinal amacrine cell, photoreceptor, and bipolar cell (n=5). Scale bar, 100 μm. GCL, ganglion cell layer; INL, inner nuclear layer; ONL, outer nuclear layer; Scr, scramble shRNA. *P<0.05.

Figure S2: cZRANB1 knockdown indirectly regulates RGC function in vitro

(A) RGCs were transfected with scramble (Scr) shRNA, cZRANB1 shRNA1, cZRANB1 shRNA2, or left untreated (Ctrl) for 24 h. qRT-PCRs were performed to detect cZRANB1 expression (n=4, *P<0.05 versus Ctrl). (B) Cell viability was detected by MTT method. The data was expressed as the relative change compared with Ctrl group (n=4, *P<0.05 versus Ctrl). (C) Ki67 staining was used to detect cell proliferation (n=4, *P<0.05 versus Ctrl group). Scale bar: 20 μm. (D) RGCs were co-cultured with wild-type Müller cells (Ctrl), Scr shRNA-transfected Müller cells, cZRANB1 shRNA1-transfected Müller cells, cZRANB1 shRNA2-transfected Müller cells, and then exposed to glutamate (3 mM) for 24 h. PI staining and quantitative analysis was performed to detect apoptotic RGCs (n=4, *P<0.05 versus Ctrl group).

Figure S3 : cZRANB1-miR-217-RUNX2 crosstalk occurs in Müller cells

(A) Müller cells were transfected with miR-217 mimic, scramble (Scr) miRNA mimic, or left untreated (Ctrl) for 24 h. qRT-PCRs were conducted to detect RUNX2 expression (n=4, *P<0.05). (B) Müller cells were co-transfected LUC-RUNX2 with or without miR-217 mimic or Scr miRNA

mimic. Luciferase activity was detected by the dual luciferase assay 24 h after transfection (n=4, *P<0.05). (C) Müller cells were transfected with Scr shRNA, cZRANB1 shRNA, or left untreated (Ctrl) for 24 h. qRT-PCRs were conducted to detect RUNX2 expression (n=4, *P<0.05). (D) Müller cells were treated as shown for 24 h. Ki67 staining and quantitative analysis was conducted to detect cell proliferation. *P<0.05 versus Ctrl group; $^{\#}P$<0.05 cZRANB1 shRNA versus cZRANB1 shRNA+RUNX2.

Supplementary methods

Quantitative real-time polymerase chain reaction (qRT-PCR)

Total RNAs were isolated and purified using the TRIzol reagent (Invitrogen). They were then reversely transcribed using the PrimeScript RT reagent Kit (TaKaRa, Dalian, China) according to the manufacturer's protocols. Gene expression was determined by the SYBRGreen PCR kit (TaKaRa).

MTT assay

Cell viability was determined by 3-(4, 5-dimethylthiazol-2-yl)-2, 5-diphenyltetrazolium bromide (MTT) assay. Cells were plated into 96-well plates overnight. After the required treatment, they were incubated with MTT solution (5mg/ml; sigma-Aldrich) for 3 h at 37°C. After MTT solution was removed, the formazan crystals were dissolved by DMSO solution. The absorbance was determined by a microplate reader (Molecular Devices) at 570 nm wavelength.

Cell proliferation assay

Cells proliferation were conducted by Ki67 staining. Briefly, glial cells were fixed with 4% PFA, permeabilized with 0.25% Triton X-100, and then blocked with 5% BSA in PBS. They were

incubated with Ki67 antibody overnight at 4°C, and then incubated with the secondary antibody for 3 h at room temperature. Finally, they were stained with DAPI to show cell nuclei.

Dual-luciferase activity assay

The 3'-UTR mRNA sequences of RUNX2 or the full sequence of cZRANB1 were cloned into the pGL3 vectors. Then, the cells were seeded in a 48-well plate and co-transfected with miRNA mimic or control mimic, pGL3-RUNX2 or pGL3-cZRANB1, and a Renilla luciferase expression construct pRL-TK (Promega, WI) using Lipofectamine 2000. Luciferase activity was detected with the dual-luciferase reporter assay system after 24 h transfection, the relative luciferase activity was calculated as the ratio of Firefly luciferase activity versus Renilla luciferase activity.

Cell apoptosis assay

Cells apoptosis were detected by propidium iodide (PI) staining. Briefly, the cells were seeded in 96-well plates and washed in PBS. They were fixed in 4% formaldehyde for 15 min, stained with PI (10 μmol/l) for 15 min, and then stained with DAPI (0.5 μg/ml) to show cell nuclei.

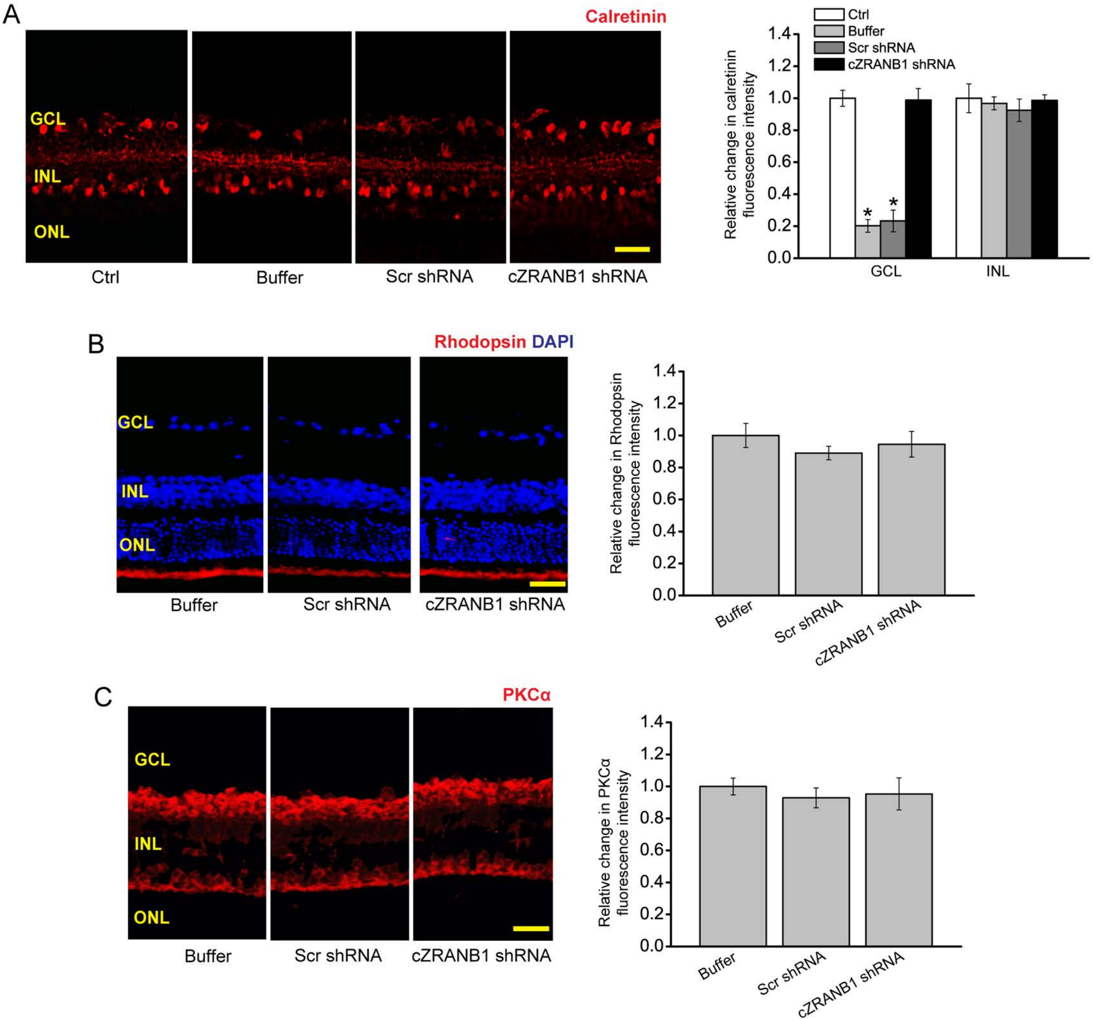

A
Calretinin
GCL
INL
ONL
Ctrl
Buffer
Scr shRNA
cZRANB1 shRNA
Relative change in calretinin fluorescence intensity
1.4
1.2
1.0
0.8
0.6
0.4
0.2
0.0
*
*
GCL
INL
B
Rhodopsin DAPI
GCL
INL
ONL
Buffer
Scr shRNA
cZRANB1 shRNA
Relative change in Rhodopsin fluorescence intensity
C
PKCα
GCL
INL
ONL
Buffer
Scr shRNA
cZRANB1 shRNA
Relative change in PKCα fluorescence intensity

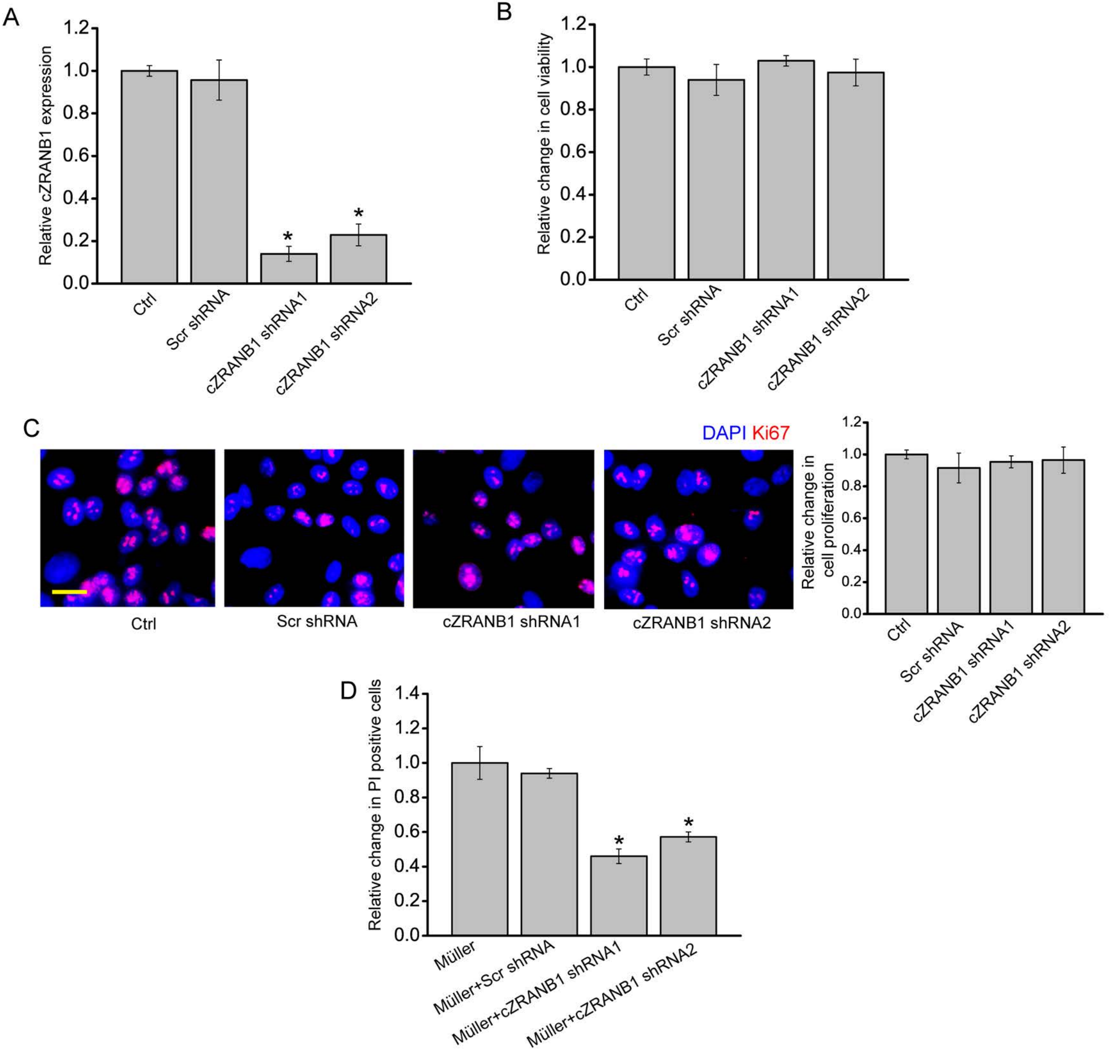
A
Relative cZRANB1 expression
1.2
1.0
0.8
0.6
0.4
0.2
0.0
*
*
Ctrl
Scr shRNA
cZRANB1 shRNA1
cZRANB1 shRNA2
B
Relative change in cell viability
1.2
1.0
0.8
0.6
0.4
0.2
0.0
Ctrl
Scr shRNA
cZRANB1 shRNA1
cZRANB1 shRNA2
C
DAPI Ki67
Ctrl
Scr shRNA
cZRANB1 shRNA1
cZRANB1 shRNA2
Relative change in cell proliferation
1.2
1.0
0.8
0.6
0.4
0.2
0.0
Ctrl
Scr shRNA
cZRANB1 shRNA1
cZRANB1 shRNA2
D
Relative change in PI positive cells
1.4
1.2
1.0
0.8
0.6
0.4
0.2
0.0
*
*
Müller
Müller+Scr shRNA
Müller+cZRANB1 shRNA1
Müller+cZRANB1 shRNA2

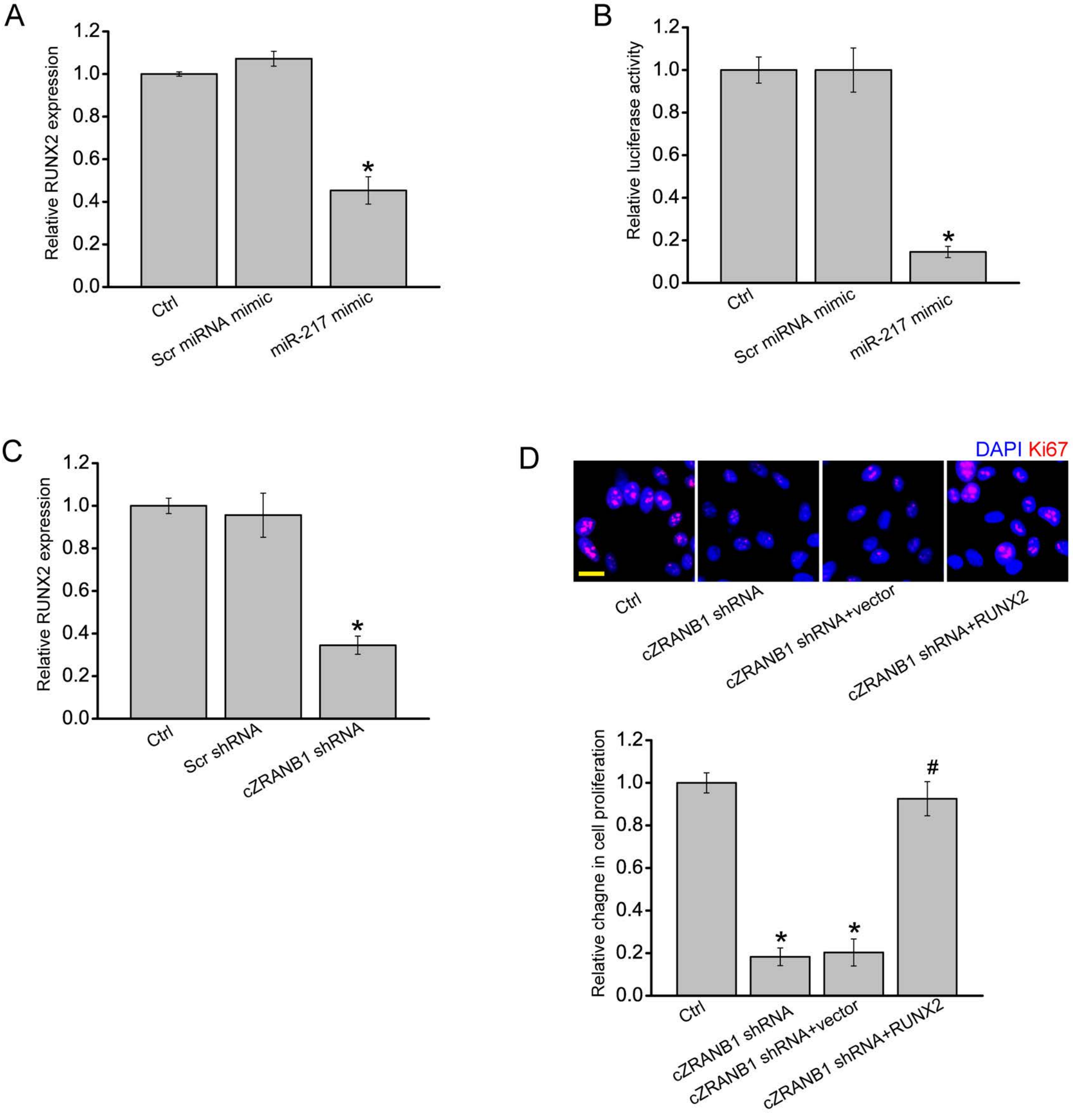
A
Relative RUNX2 expression
1.2
1.0
0.8
0.6
0.4
0.2
0.0
*
Ctrl
Scr miRNA mimic
miR-217 mimic
B
Relative luciferase activity
1.2
1.0
0.8
0.6
0.4
0.2
0.0
*
Ctrl
Scr miRNA mimic
miR-217 mimic
C
Relative RUNX2 expression
1.2
1.0
0.8
0.6
0.4
0.2
0.0
*
Ctrl
Scr shRNA
cZRANB1 shRNA
D
DAPI Ki67
Ctrl
cZRANB1 shRNA
cZRANB1 shRNA+vector
cZRANB1 shRNA+RUNX2
Relative chagne in cell proliferation
1.2
1.0
0.8
0.6
0.4
0.2
0.0
*
*
#
Ctrl
cZRANB1 shRNA
cZRANB1 shRNA+vector
cZRANB1 shRNA+RUNX2
